# Radio Frequency Identification Temperature/CO_2_ Sensor Using Carbon Nanotubes

**DOI:** 10.3390/nano13020273

**Published:** 2023-01-09

**Authors:** Ayesha Habib, Safia Akram, Mohamed R. Ali, Taseer Muhammad, Sajeela Zainab, Shafia Jehangir

**Affiliations:** 1 Department of Electrical Engineering, MCS, National University of Sciences and Technology, Islamabad 44000, Pakistan; 2 Department of Basic Sciences, MCS, National University of Sciences and Technology, Islamabad 44000, Pakistan; 3 Center of Research, Faculty of Engineering and Technology, Future University in Egypt, New Cairo 11835, Egypt; 4 Department of Mathematics, College of Science, King Khalid University, Abha 61413, Saudi Arabia; 5 ACTSENA Research Group, University of Engineering and Technology, Taxila 47080, Pakistan

**Keywords:** inkjet-printed electronics, chipless tag, cross-section curve, backscattering, sensor

## Abstract

In the world of digitization, different objects cooperate with the Internet of Things (IoT); these objects also amplify using sensing and data processing structures. Radio frequency identification (RFID) has been identified as a key enabler technology for IoT. RFID technology has been used in different conventional applications for security, goods storage, transportation and asset management. In this paper, a fully inkjet-printed chipless radio frequency identification (RFID) sensor tag is presented for the wireless identification of tagged objects. The dual polarized tag consists of two resonating structures functioning wirelessly. One resonator works for encoding purpose and other resonator is used as a CO_2_/temperature sensor. The sensing behavior of the tag relies on the integration of a meandered structure comprising of multi-wall carbon nanotubes (MWCNT). The MWCNT is highly sensitive to CO_2_ gas. The backscattered response of the square-shaped cascaded split ring resonators (SRR) is analyzed through a radar cross-section (RCS) curve. The overall tag dimension is 42.1 mm × 19.5 mm. The sensing performance of the tag is examined and optimized for two different flexible substrates, i.e., PET and Kapton^®^HN. The flexible tag structure has the capability to transmit 5-bit data in the frequency bands of 2.36–3.9 GHz and 2.37–3.89 GHz, for PET and Kapton^®^HN, respectively. The proposed chipless RFID sensor tag does not require any microchip or a power source, so it has a great potential for low-cost and automated temperature/CO_2_ sensing applications.

## 1. Introduction

Over the past few years, the Internet of Things (IoT) has played a significant role and improving the quality of lives in different domains [1]. Due to its affordability, increased performance efficiency, compact size and high data capacity, it is being deployed in various applications, such as healthcare, transportation, logistics, supply chain, vehicle identification, baggage tracking in airports and other industrial applications [2]. The IoT permits physical objects to perform their tasks by coordinating and communicating with each other to share information [3]. The IoT renovates the physical objects from traditional to smart by manipulating their underlying architecture, applications and protocols [4]. Generally, IoT uses wireless technologies and allows the automation of everything around us [5].

The concept of the IoT is that it can be instigated using radio frequency identification (RFID) technology [6]. Radio frequency identification (RFID) is a widespread technology across the globe, using electromagnetic (EM) waves from the reader to remotely detect an object with complete accuracy [7,8]. The RFID system provides ease in detection as well as longer read ranges [9,10]. RFID tags can be classified into three categories: active RFID tags, semi-passive RFID tags, and passive RFID tags [11]. Active tags are chipped (ASIC-based) RFID tags which depend on external power sources to function [12]. Despite the efforts of manufacturers, active tags are still very costly to be utilized for large-scale deployment [13]. In semi-passive RFID tags, to initiate a response from the tag, a signal from the reader is used. The semi-passive tag also used a battery, but it is distinct from the active tags [14]. The use of a battery in such tags is to power a sensor and run the integrated circuit on the chip, and not for generating a response. Passive RFID tags do not require any internal power source but are powered by electromagnetic waves transmitted from the RFID reader [15]. The lower price of such tag makes the RFID system cost-effective for many industries [16].

RFID tags are the main focus of researchers [7,17]. They are powered by the EM field from the reader and duly reliant upon the backscattered EM response. Passive tags offer high reliability and extended lifetime. Moreover, a maintenance cost of almost zero makes them highly affordable for a large-scale production. The only limitation of passive tags is their limited read range compared to the active tags [8]. Passive chipless RFID tags are further classified as: backscattering-based tags and retransmission-based tags. The signal retransmission-based tags comprise of resonating structures along with transmission line. Cross-polarized transmitting and receiving antennas are used for signal transmission and reception. The backscattering-based tags are loaded with simple resonating structure having compact size [18].

Carbon nanotube (CNT)-based integrated sensors attribute indomitable sensitivity to stimuli, depicting a quick response to variations in surrounding environment. Highly sensitive regions of operations with coarse environments [8], prone to dangerous chemicals, gases or temperature variations [19], require inexpensive approach to keep track of physical parameters at volumetric level. In comparison with the wired sensing networks, chipless RFID sensors provide flexibility in the ease of installation and operation. Chipless sensors operate by continuously monitoring the backscattered EM response of the tag for the same interrogating signal [8,17,18]. The passive sensor-based tags may consist of sensitive materials such as PVA (polyvinyl alcohol) [20], Stanyl^®^, Zenite^®^, Zytel^®^, Valox^®^, etc. [21], for sensing along with data-encoding conductive strips to eliminate the need for any discrete sensor component. Such sensors can easily be realized by printing techniques on flexible laminates, thus cutting down per unit cost of tag [22,23]. The humidity sensing tested for single-layer silver ink-based resonators printed on flexible laminate, i.e., Kapton^®^ HN, is presented in [24]. The transmission line-based conductive tracks printed on hygroscopic material are tested for various humidity levels.

In this paper, a novel fully printable RFID-enabled temperature sensing infrastructure is presented. Inkjet printing is an efficient and faster way to print electronics on flexible substrates. The proposed tag is realized on flexible substrate materials using silver nanoparticle-conductive traces, which makes it suitable for low-cost mass production. The carbon nanotubes are used to observe the sensing behavior of the tag. The paper is organized as follows: Section 2 describes the backscattering phenomenon of proposed chipless RFID tag. Section 3 defines the software tool and the geometric configuration of the proposed tag. Section 4 designates the experimental outcomes and discuss the graphical results of proposed tag and finally, Section 5 involves of conclusion with future work.

## 2. Materials and Methods

**Figure 1:**
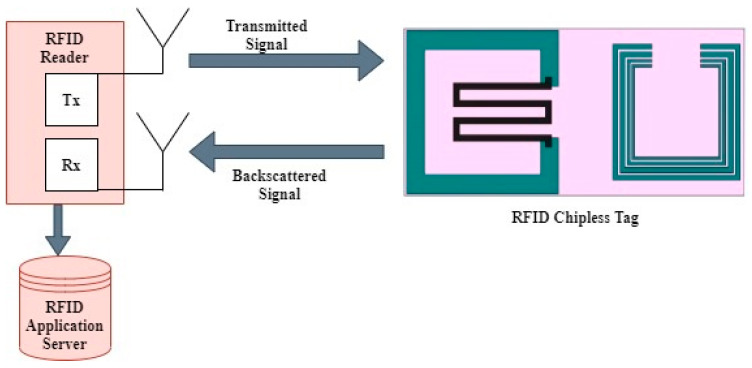
Proposed RFID Setup.

Contrary to conventional RFID tags, chipless tags do not require a transmission protocol to perform the transmitting/receiving operation without an IC chip [7]. The operational principle of chipless RFID tags is based on the backscattering phenomenon [25]. In Figure 1, the backscattering phenomenon is shown, which consists of an interrogator, also known as reader, and a transponder defined as a chipless RFID tag. The reader transmits the EM wave and the chipless RFID tag absorbs the transmitted waves, which simulates current on the surface of the tag. After absorbing the EM wave, a modulated signal is produced that acts as a backscattered signal towards the reader. The backscattered signal contains the encoded data, and the received information is identified using a unique tag ID [26]. The reader transmits an incident plane wave as an interrogation signal towards the tag and, in response, a backscattered signal containing the tag information is sent to the reader. After processing the received signal, 5-bit encoded data are produced. The change in gas concentration/temperature is observed through the backscattered response. Due to the change in surrounding environmental conditions, the result reflects the peak amplitude shift in the sensing bit response, whereby there is no peak amplitude shift in the neighboring encoding bits [8], while keeping the same resonating frequencies for both sensing and encoding bits.

**Figure 2:**
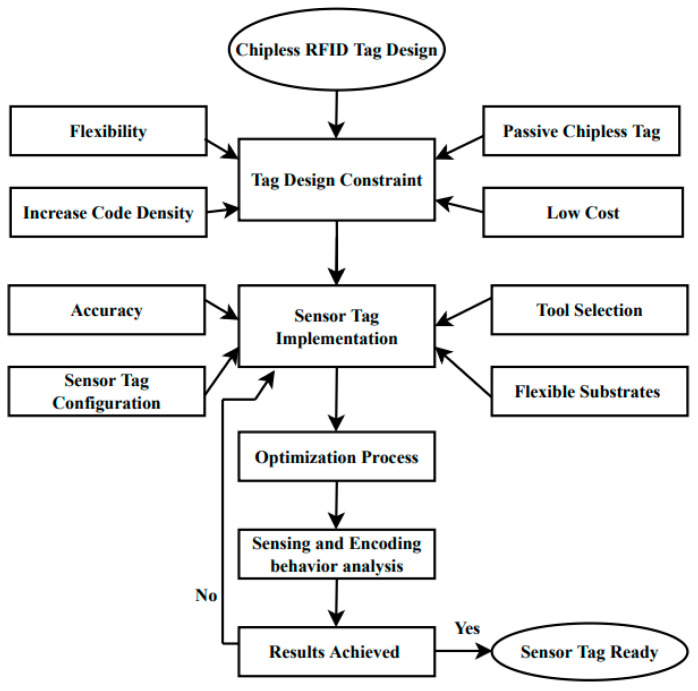
Flow chart.

The tag structure was designed and analyzed in CST STUDIO SUITE^®^. To measure the RCS response of tag, the tag is placed at a far-field distance from the reader. The radar cross section (RCS) response of the tag is determined at a Fraunhofer far-field distance using Equation (1).

(1) $$R\;=2D^{2}/\lambda$$

where *R* is the far-field distance, *D* is the largest dimension of the tag, and *λ* is the wavelength [27]. The power that is broadcasted by the transmitting antenna is received by the receiving antenna. Using Friis transmission equation, we find the transmitting power.

(2) $$\frac{P_{\mathrm{RX}}}{P_{\mathrm{TX}}}={\left(\frac{\lambda }{4r\pi }\right)}^{2}G_{\mathrm{TX}}G_{\mathrm{RX}}$$

where the distance between transmitter and receiver is defined as *r*, *G*_TX_ is the gain of the transmitting antenna and *G*_RX_ is the gain of receiving antenna, *λ* is the wavelength, and *P*_TX_ and *P*_RX_ are the transmitted and received power [28]. For proper RFID operation, all the tagged objects should be placed in the read range of the RFID system [29]. For the RFID system, the maximum read range could be determined by Equation (3) [30].

(3) $$R^{\max}=\sqrt[{4}]{P_{\mathrm{TX}}G_{\mathrm{TX}}G_{\mathrm{RX}}\frac{\lambda^{2}}{{\left(4\pi \right)}^{3}P_{\mathrm{RX}}}\sigma_{\min}}$$

where *P*_TX_ is transmitting power, *G*_TX_ is transmitting antenna gain, *G*_RX_ is receiving antenna gain, *λ* is the wavelength, *P*_RX_ is the sensitivity of the receiver and *σ*_min_ is the most minimum RCS level that can be detected by the reader. The proposed approach that is presented in this paper to develop a chipless RFID sensor tag, which is explained in the flow chart, as shown in Figure 2.

**Figure 3:**
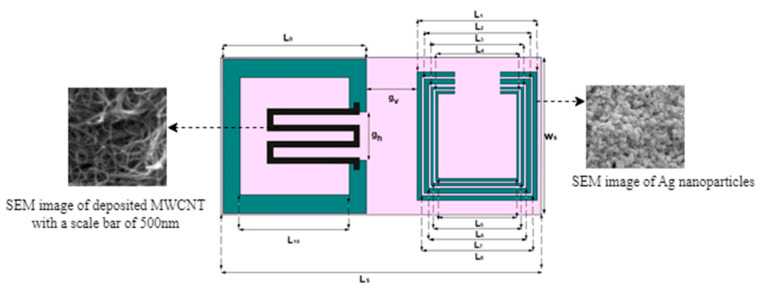
Proposed Tag Dimension and SEM Image of MWCNT.

As presented in Figure 3, the tag is loaded with one sensing resonator and multi-resonators for encoding purpose. The gaps (g_v_,g_h_) shown in Figure 3, between the sensing part and encoding part, are 7 mm to reduce the coupling effect between them. Every resonator corresponds in a one-to-one relation with its respective data bit. All the resonators have different lengths, producing peaks at different resonating frequencies. A tradeoff is made between the bandwidth of each peak response, size of resonators and magnitude of EM response to optimize the tag design. The detailed parameters of the proposed optimized tag are elaborated in the Table 1. The square SRR are printed on Kapton^®^HN (ε_r_ = 3.5, tanδ = 0.0026) laminate sheet. Using DMP2800 inkjet printer, nanoparticle-based silver ink (Cabot Ink CCI-300) is deposited upon the substrate to make conductive arms/strips. The conductivity of silver nanoparticle-based ink is 9 × 10^6^ S/m, with a thickness of 0.015 mm. However, the silver nanoparticle-based ink has a very low conductivity compared to bulk conductors such as copper metal, but their production of lightweight tags has made them a competent solution to be deployed for low-cost applications.

To avoid performance degradation, the width of sensing resonator is 2 mm. The gap between the arms of the sensor part provides a space to insert a multi-wall carbon nanotube (MWCNT)-based sensitive strip, i.e., meandered structure. This increases the area of the sensing resonator and makes it more prone to environmental changes. The SEM image of MWCNT at 2 µm resolution is shown in Figure 3. The initial sheet resistance of MWCNT is 1000 Ω/sq at 25 °C.

**Figure 4:**
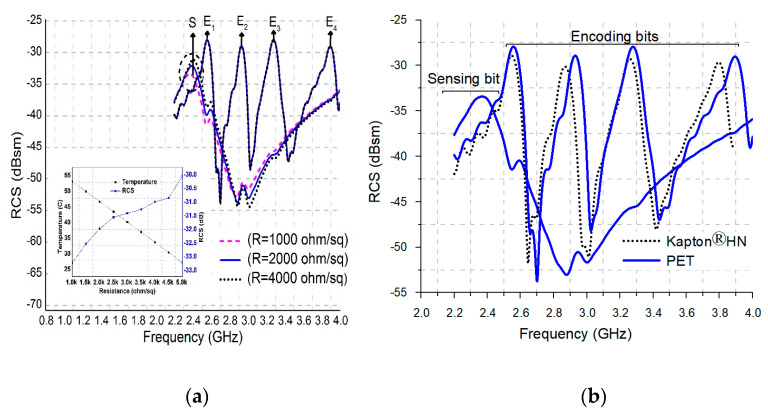
(**a**) Sensing behavior, (**b**) comparison graph.

The proposed temperature/CO_2_ sensor tag is tested for sensing by incorporating MWCNT into the tag’s structure. A homogeneous solution of CNT is obtained by mixing a powdered MWCNT with water. However, water is not an excellent choice to be choose as a solvent compared to other polymers such as ethylene glycol [31,32], but keeping in view the climatic requirement and manufacturing cost, water-based CNT can be used. At room temperature, the MWCNT solution is injected precisely over the sensing slot structure of the tag. The dropped MWCNT over the tag starts absorbing the CO_2_ gas. The resistivity of MWCNT is directly proportional to the absorbing concentration of CO_2_ gas. The frequency response of the sensing slot starts deteriorating with increased absorption of CO_2_ gas, as shown in Figure 4a.

## 3. Results

The proposed tag design is a novel, compact-sized, low-cost, chipless RFID tag with integrated sensors. The novelty of the proposed work consists of different lengths for sensor and encoders, leading to different resonant frequencies for them. Hence, with this optimization, the sensing and encoding bits are analyzed simultaneously on the same RCS curve. It has a dual-polarized split ring resonator (SRR)-based structure, in which sensor and encoder conducting tracks are orthogonal to each other. The resonator-depicting sensor bit is vertically polarized, excited by vertically polarized plane waves. The conducting tracks/strips producing encoding bits other than sensing bits are horizontally polarized and the yield response when horizontally polarized plane wave is sent to them. Hence, this dual-polarized nature of the tag helps in isolating the sensing response from the reference-encoding signal. The tag response, as an integrated sensor, is continually observed as RCS response.

The proposed passive chipless RFID sensor tag has a five-bit code density and can produce 2^5^ = 32 unique data combinations. The optimized tag configuration has high-quality resonators inside a miniaturized tag design. The dual-polarized sensor tag provides the benefit of orientation independence along with the elimination of polarization alignment. The MWCNTs are also sensitive to other gases such as oxides of carbon and nitrogen, etc.

Figure 4a shows the optimized results for different resistance values, i.e., 1000 Ω/sq, 2000 Ω/sq and 4000 Ω/sq. The sensing bit peak response shows a rise in amplitude with an increase in resistance of MWCNT. The variation in RCS response does not affect the resonant frequencies, for both sensing and encoding data bits. The results validate that with change in the sensed parameters, only the amplitude of the sensing bit shows variation, while the peak amplitude of neighboring encoding bits remain unchanged. The temperature varies linearly with respect to the sheet resistance.

The larger the resistance, the less power resides inside the MWCNTs and the conductive strips reflect larger EM energy back toward the reader. Moreover, a parametric study is duly carried out to optimize the sensor performance using two different flexible laminates. Several tag prototypes have been realized to validate the reliability parameter.

Figure 4b shows a comparison between Kapton^®^HN and PET substrates. In Figure 4b, it is clearly shown that the substrate has a slight effect on the peak responses of the sensing bit, as well as encoding bits. The frequency band for Kapton ranges is between 3.8 and 2.28 GHz and for PET ranges is between 3.9 and 2.36. Furthermore, it has been analyzed that the tag design, optimized for flexible Kapton^®^HN substrate, strives for efficient band utilization.

## 4. Discussion

**Figure 5:**
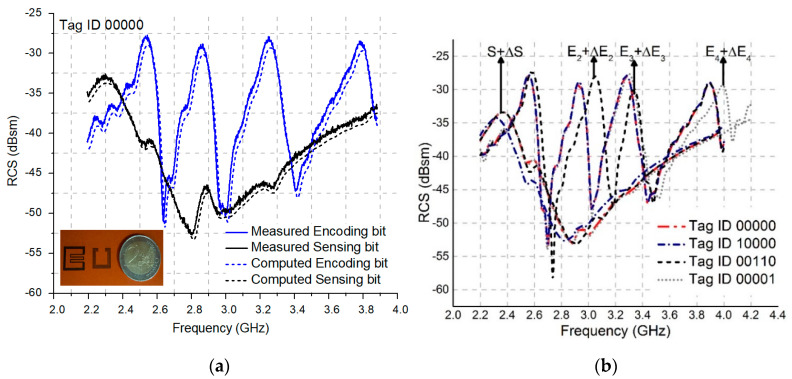
(**a**) Measured and computed RCS response, (**b**) measured tag IDs.

The measured and computed response of the tag along with the prototype is presented in Figure 5a, where the measured tag response reflects close correspondence to the computed simuations.

Originally, the resonance peaks produced 00000 data words as a reference tag ID. Using the frequency shifting phenomena for encoding purpose, changing the length of specific resonating structure(s) by a factor of ’∆’, changes the logic state to 1. The resonance peaks with no shifts correspond to logic state-0. Logic state-1 represents ‘1’, whereas logic state-0 represents ‘0’. Following this technique, multiple tag IDs are generated as shown in Figure 5b.

**Figure 6:**
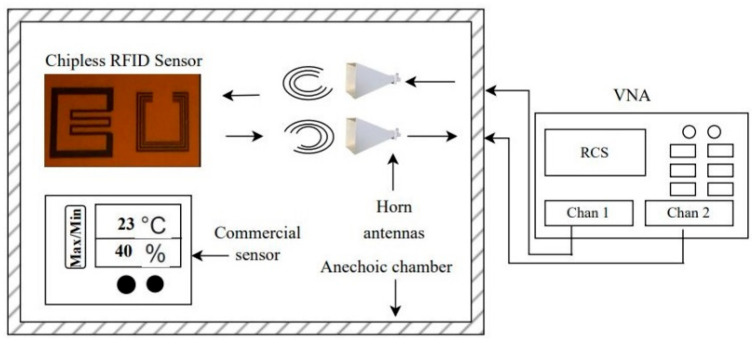
A schematic of test setup.

The sensor tag is tested in an environmental chamber inspired by N. Javed et al. [31]. The schematic of test setup is shown in Figure 6. The test chamber comprises two horn antennas of gain = 14 dB, a chipless sensor tag, an electronic sensor and a vector network analyzer (VNA) R&S^®^ZVL13. The climatic chamber is exploited for the temperature/CO_2_ sensing attribute of the tag by injecting 20,000 ppm CO_2_ gas concentration. The sensor tag is mounted onto the surfaces, i.e., wooden plank or cardboard, as having materials beneath these thin substrates (such as Kapton^®^HN and PET) isolate the tag from strong external influences.

**Figure 7:**
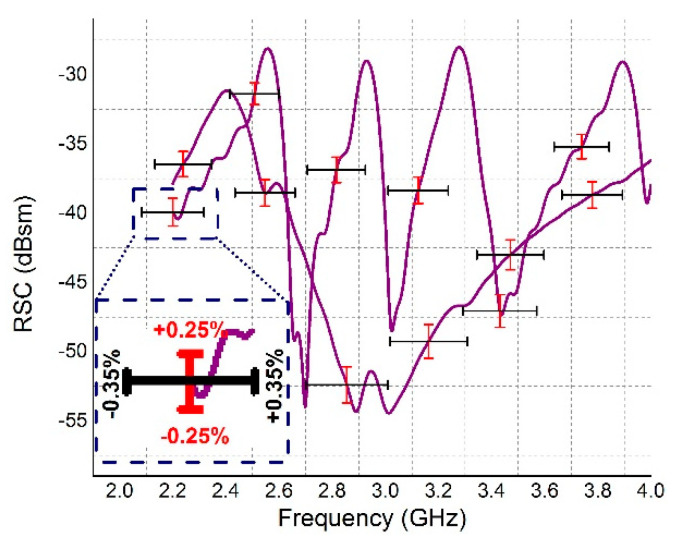
Reliability curve.

To analyze the reliability of the tag, five protypes of the sensor tag were manufactured and tested. The reliability performance of the sensor tag is shown in Figure 7. It has been analyzed that the tag has tolerance of 0.25% and 0.35% in the RCS and frequency band, respectively, which are of interest. The parametric conclusion drawn is presented in Table 2.

## 5. Conclusions

In this paper, a fully printable, chipless RFID temperature/CO_2_ sensor tag is presented. The firm agreement between the experimental and computed simulations assisted in validating the tag design. The integrated sensors exploit the backscattering principle using square split ring resonators, to simultaneously sense and encode the data bits. The tag has the capability to transmit 2^5^ = 32 unique data combinations. Due to its flexible nature, the tag could be the best choice to be deployed on curved/irregular surfaces made of wooden planks or cardboard. The miniaturized chipless RFID sensor tag, with its lightweight feature, can be used in various low-cost sensing applications.

## Figures and Tables

**Table 1:** Optimized tag dimensions.

Parameters	Value (mm)	Parameters	Value (mm)
Ls	42.10	L_5_	10.50
Ws	19.50	L_6_	11.60
L_1_	15.90	L_7_	12.90
L_2_	14.10	L_8_	14.70
L_3_	12.30	L_9_	18.50
L_4_	11.10	L_10_	14.50
g_h_	7.00	g_v_	7.00

**Table 2:** Comparison of substrates.

Substrate	Kapton^®^HN	PET
Thickness (mm)	0.125	0.100
Frequency (GHz)	3.8–2.28	3.9–2.36
Bandwidth (GHz)	1.52	1.54
Conducting ink	silver	silver
Flexibility	√	√

## Data Availability

Not applicable.
